# Supplementary material for: Clinician perspectives on what constitutes good practice in community services for people with complex emotional needs: A qualitative thematic meta-synthesis
Source: PLoS One. 2022 May 5;17(5):e0267787. doi: 10.1371/journal.pone.0267787 (PMC9070883; doi:10.1371/journal.pone.0267787)
Supplement: S1 Table — (DOCX) [file pone.0267787.s002.docx]

**S1 Table. Quality assessment according to the Critical Appraisal Skills Programme**

| First Author, Year. | 1. Was there a clear statement of the aims of the research? | 2. Is a qualitative methodology appropriate? | 3. Was the research design appropriate to address the aims of the research? | 4. Was the recruitment strategy appropriate to the aims of the research? | 5. Was the data collected in a way that addressed the research issue? | 6. Has the relationship between researcher and participants been adequately considered? | 7. Have ethical issues been taken into consideration? | 8. Was the data analysis sufficiently rigorous? | 9. Is there a clear statement of findings? | 10. How valuable is the research? |
| --- | --- | --- | --- | --- | --- | --- | --- | --- | --- | --- |
| Bosanac, 2015 [1] | Yes | Yes | Yes | Can’t Tell | Yes | No | No | Can’t Tell | Yes | Unclear |
| Carmel, 2014 [2] | Yes | Yes | Yes | Yes | Yes | No | No | Can’t Tell | Yes | Valuable |
| Crawford, 2007a [3] | Yes | Yes | Yes | Yes | Yes | No | Yes | Yes | Yes | Valuable |
| Crawford, 2007b [4] | * |  |  |  |  |  |  |  |  |  |
| Crawford, 2010 [5] | Yes | Yes | Yes | Yes | Yes | No | Yes | Yes | Yes | Valuable |
| Donald, 2017 [6] | Yes | Yes | Yes | Can’t Tell | Yes | No | Can’t tell | Yes | Yes | Valuable |
| Fanaian, 2013 [7] | Yes | Yes | Yes | Can’t Tell | Can’t Tell | No | Yes | Yes | Yes | Valuable |
| French, 2019 [8] | Yes | Yes | Yes | Yes | Yes | No | Can’t Tell | Yes | Yes | Valuable |
| Herschell, 2009 [9] | No | Yes | Yes | Yes | Yes | No | Yes | Yes | Yes | Valuable |
| Hogard, 2010 [10] | Yes | Yes | Yes | Can’t Tell | Can’t Tell | No | No | Can’t Tell | Yes | Unclear |
| Hutton, 2017 [11] | Yes | Yes | Yes | Yes | Yes | No | Yes | Yes | Yes | Valuable |
| Koekkoek, 2009 [12] | Yes | Yes | Yes | Yes | Yes | No | No | Yes | Yes | Valuable |
| Lamph, 2019 [13] | Yes | Yes | Yes | Yes | Yes | No | Can’t Tell | Yes | Yes | Valuable |
| Langley, 2005 [14] | Yes | Yes | Yes | Can’t Tell | Yes | No | Yes | Yes | Yes | Valuable |
| Lee, 2008 [15] | No | Can’t Tell | Can’t Tell | Can’t Tell | Can’t Tell | No | No | Can’t Tell | No | Unclear |
| Morant, 2003 [16] | Yes | Yes | Yes | Yes | Yes | No | Can’t Tell | Can’t Tell | Yes | Valuable |
| O’Connell, 2013 [17] | Yes | Yes | Yes | Can’t Tell | Can’t Tell | No | Yes | Can’t Tell | Yes | Valuable |
| Perseius, 2003 [18] | Yes | Yes | Yes | Yes | Yes | Yes | Yes | Yes | Yes | Valuable |
| Perseius, 2007 [19] | Yes | Yes | Yes | Yes | Yes | No | Yes | Yes | Yes | Valuable |
| Pigot, 2019 [20] | Yes | Yes | Yes | Yes | Yes | No | Yes | Yes | Yes | Valuable |
| Priest, 2011 [21] | Yes | Yes | Yes | Can’t Tell | Yes | No | Can’t Tell | Yes | Yes | Valuable |
| Rizq, 2012 [22] | Yes | Yes | Yes | Yes | Yes | Yes | Yes | Yes | Yes | Valuable |
| Stalker, 2005 [23] | Yes | Yes | Yes | Can’t Tell | Yes | No | Yes | Yes | Yes | Valuable |
| Stroud, 2013 [24] | Yes | Yes | Yes | Can’t Tell | Yes | Yes | Yes | Yes | Yes | Valuable |
| Sulzer, 2016 [25] | Yes | Yes | Yes | Yes | Yes | No | Yes | Yes | Yes | Valuable |
| Thompson, 2008 [26] | Yes | Yes | Yes | Yes | Yes | No | Yes | Yes | Yes | Valuable |
| Vyas, 2017 [27] | Yes | Yes | Yes | Can’t Tell | Yes | Yes | Can’t Tell | Yes | Yes | Valuable |
| Wilson, 2018 [28] | Yes | Yes | Yes | Yes | Yes | No | Can’t Tell | Yes | Yes | Valuable |
| Wlodarczyk, 2018 [29] | Yes | Yes | Yes | Yes | Yes | No | Yes | Yes | Yes | Valuable |

*Crawford2007b is a short published paper based on Crawford 2007a which is a long-form report and provides details in full. Only the quality ratings of Crawford 2007a have therefore been provided.

1. Bosanac P, Hamilton B, Beatson J, Trett R, Rao S, Mancuso S, et al. Mentalization-based intervention to recurrent acute presentations and self-harm in a community mental health service setting. *Australasian Psychiatry*. 2015;23(3):277-81.

2. Carmel A, Rose ML, Fruzzetti AE. Barriers and solutions to implementing dialectical behavior therapy in a public behavioral health system. *Adm Policy Ment Health*. 2014;41(5):608-14.

3. Crawford M, Rutter D, Price K, Weaver T, Josson M, Tyrer P, et al. Learning the lessons: a multi-method evaluation of dedicated community-based services for people with personality disorder. *London: National Co-ordinating Centre for NHS Service Delivery & Organisation*. 2007.

4. Crawford M, Rutter D. Lessons Learned from an Evaluation of Dedicated Community-based Services for People with Personality Disorder. *The Mental Health Review*. 2007;12(4):55-61.

5. Crawford MJ, Adedeji T, Price K, Rutter D. Job Satisfaction and Burnout Among Staff Working in Community-Based Personality Disorder Services. *International Journal of Social Psychiatry*. 2010;56(2):196-206.

6. Donald F, Duff C, Lawrence K, Broadbear J, Rao S. Clinician perspectives on recovery and borderline personality disorder. *The Journal of Mental Health Training, Education and Practice*. 2017;12(3):199-209.

7. Fanaian M, Lewis KL, Grenyer BFS. Improving services for people with personality disorders: Views of experienced clinicians. *International Journal of Mental Health Nursing*. 2013;22(5):465-71.

8. French L, Moran P, Wiles N, Kessler D, Turner KM. GPs’ views and experiences of managing patients with personality disorder: a qualitative interview study. *BMJ Open* [Internet]. 2019; 9(2):e026616. DOI: 10.1136/bmjopen-2018-026616

9. Herschell AD, Kogan JN, Celedonia KL, Gavin JG, Stein BD. Understanding Community Mental Health Administrators' Perspectives on Dialectical Behavior Therapy Implementation. *Psychiatric Services*. 2009;60(7):989-92.

10. Hogard E, Ellis R. An evaluation of a managed clinical network for personality disorder: breaking new ground or top dressing? *Journal of Evaluation in Clinical Practice*. 2010;16(6):1147-56.

11. Hutton R, Hodge S, Tighe M. Switching roles: a qualitative study of staff experiences of being dialectical behaviour therapists within the National Health Service in England. *The Cognitive Behaviour Therapist* [Internet]. 2017; 10:e6. DOI: 10.1017/S1754470X17000083

12. Koekkoek B, van Meijel B, Schene A, Hutschemaekers G. Clinical Problems in Community Mental Health Care for Patients with Severe Borderline Personality Disorder. *Community Mental Health Journal*. 2009;45(6):508.

13. Lamph G, Baker J, Dickinson T, Lovell K. Personality disorder co-morbidity in primary care ‘Improving Access to Psychological Therapy’ services: A qualitative study exploring professionals' perspectives of working with this patient group. *Personality and Mental Health*. 2019;13(3):168-79.

14. Langley GC, Klopper H. Trust as a foundation for the therapeutic intervention for patients with borderline personality disorder. *Journal of Psychiatric and Mental Health Nursing*. 2005;12(1):23-32.

15. Lee T, McLean D, Moran P, Jones H, Kumar A. A pilot personality disorder outreach service: development, findings and lessons learnt. *Psychiatric Bulletin*. 2008;32(4):127-30.

16. Morant N, King J. A multi-perspective evaluation of a specialist outpatient service for people with personality disorders. *The Journal of Forensic Psychiatry & Psychology*. 2003;14(1):44-66.

17. O’Connell B, Dowling M. Community psychiatric nurses’ experiences of caring for clients with borderline personality disorder. *Mental Health Practice*. 2013;17(4):27-33.

18. Perseius K-I, Öjehagen A, Ekdahl S, Åsberg M, Samuelsson M. Treatment of suicidal and deliberate self-harming patients with borderline personality disorder using dialectical behavioral therapy: the patients’ and the therapists’ perceptions. *Archives of Psychiatric Nursing*. 2003;17(5):218-27.

19. Perseius KI, Kåver A, Ekdahl S, Åsberg M, Samuelsson M. Stress and burnout in psychiatric professionals when starting to use dialectical behavioural therapy in the work with young self-harming women showing borderline personality symptoms. *Journal of Psychiatric and Mental Health Nursing*. 2007;14(7):635-43.

20. Pigot M, Miller CE, Brockman R, Grenyer BFS. Barriers and facilitators to the implementation of a stepped care intervention for personality disorder in mental health services. *Personality and Mental Health*. 2019;13(4):230-8.

21. Priest P, Dunn C, Hackett J, Wills K. How can mental health professionals best be supported in working with people who experience significant distress? *Journal of Mental Health* [Internet]. 2011; 20(6):543-54 pp.]. DOI: 10.3109/09638237.2011.577115

22. Rizq R. ‘There's always this sense of failure’: an interpretative phenomenological analysis of primary care counsellors' experiences of working with the borderline client. *Journal of Social Work Practice*. 2012;26(1):31-54.

23. Stalker K, Ferguson I, Barclay A. ‘It is a horrible term for someone’: service user and provider perspectives on ‘personality disorder’. *Disability & Society*. 2005;20(4):359-73.

24. Stroud J, Parsons R. Working with borderline personality disorder: A small-scale qualitative investigation into community psychiatric nurses' constructs of borderline personality disorder. *Personality and Mental Health*. 2013;7(3):242-53.

25. Sulzer SH, Muenchow E, Potvin A, Harris J, Gigot G. Improving patient-centered communication of the borderline personality disorder diagnosis. *Journal of Mental Health*. 2016;25(1):5-9.

26. Thompson AR, Donnison J, Warnock-Parkes E, Turpin G, Turner J, Kerr IB. Multidisciplinary community mental health team staff's experience of a ‘skills level’ training course in cognitive analytic therapy. *International Journal of Mental Health Nursing*. 2008;17(2):131-7.

27. Vyas A, Spain C, Rawlinson D. Working in a therapeutic community: exploring the impact on staff. *Therapeutic Communities: The International Journal of Therapeutic Communities*. 2017;38(1):32-40.

28. Wilson R, Weaver T, Michelson D, Day C. Experiences of parenting and clinical intervention for mothers affected by personality disorder: a pilot qualitative study combining parent and clinician perspectives. *BMC Psychiatry*. 2018;18(1):152.

29. Wlodarczyk J, Lawn S, Powell K, Crawford GB, McMahon J, Burke J, et al. Exploring General Practitioners’ Views and Experiences of Providing Care to People with Borderline Personality Disorder in Primary Care: A Qualitative Study in Australia. *International Journal of Environmental Research and Public Health*. 2018;15(12):2763.
