# Supplementary material for: Clinician perspectives on what constitutes good practice in community services for people with complex emotional needs: A qualitative thematic meta-synthesis
Source: PLoS One. 2022 May 5;17(5):e0267787. doi: 10.1371/journal.pone.0267787 (PMC9070883; doi:10.1371/journal.pone.0267787)
Supplement: S2 Table — (DOCX) [file pone.0267787.s003.docx]

**S2 Table. Table of quotes.**

| **Theme** | | **Quotes** |
| --- | --- | --- |
|  | Subtheme |  |
| **Stigma and the Use and Misuse of Diagnosis** | | |
|  |  | *“The global, all‐encompassing nature of the diagnosis, coupled with the view that it was untreatable, could have a devastating impact on the individual, while also leading to a lack of therapeutic optimism on the part of clinicians. Personality disorder was seen as having all the drawbacks of a mental illness diagnosis, especially in terms of stigma, but none of the benefits, particularly access to services. Likewise, the contested and uncertain nature of personality disorder limited the potential for users to gain some control over their condition through knowledge and information. The diagnosis could lead to people facing discrimination and stereotyping within mental health services, within generic health and social care services and within society at large, with individuals being labelled as attention‐seeking and demanding.”*  *“A different view expressed by some service providers was that personality disorder is best understood as a form of social deviance or cultural rule‐breaking. One respondent described people with a personality disorder diagnosis as those whose ‘behavior, attitudes, lifestyles seem to consistently transgress cultural norms, which brings them into conflict with other people, in the absence of any symptom of an underlying mental illness…’ These respondents believed that a diagnosis of personality disorder could simply serve to medicalize or pathologize an individual’s feelings of distress.” (Stalker et al., 2005)[1]* |
| **The Patient Journey into Services: Nowhere to Go** | | |
|  |  | *“Several GPs described adopting a strategy of ‘writing-up’, or embellishing descriptions of a patient’s risk status in order to ensure that the patient was seen by secondary care services. On the other hand, faced with an overly cautious response from IAPT, several GPs described emphasising the patient’s more ‘agreeable’ mental health conditions, such as depression or anxiety, to maximise the chances of the patient being accepted into treatment.”*  *“GPs also described patients with PD as having to endure particularly long waits, before being seen—waiting times that often far exceeded those experienced by patients with other mental health problems. Indeed, several GPs described patients with PD having to wait over 12 months for treatment. They felt shortening waiting times would reduce the likelihood of mental health problems escalating or patients disengaging from the health service altogether.” (French et al., 2019)[2]* |
|  |  | *“Unfortunately, because of the model of stepped care in the Trust, there were barriers to primary care staff wishing to refer patients directly into a tertiary service, such as a specialist psychology service for people with PD. ‘…Have a real problem in getting through the cycle of exclusion: GPs who identify suitable patients cannot refer to [tertiary psychology service], they have to refer to the CMHT and get them to take person on… so we cannot keep clients out of mental health services, and CMHTs can say they won’t take them, as don’t meet their criteria.’”*  *“The majority of referrers made reference to the assessment as an important part of the process, and some stated that having an assessment was one of the reasons for referring someone to the service. Referrers linked to two services made specific comment of how much they valued the provision of a comprehensive assessment, even if the service user was not taken on. Benefits included helping the referrer to develop their own management plan or to better understand the service user’s problems and building confidence and trust for the service user.” (Crawford et al., 2007)[3]* |
| **Therapeutic Relationships: Connection and Distance** | | |
|  |  | *“A few service provider respondents however were careful not to locate ‘the problem’ within individual service users. They believed that unhelpful responses from mental health services were often responsible for compounding people’s problems shown, for example, in judgemental attitudes expressed by some staff who reportedly used words like ‘manipulative’, ‘attention‐seeking’ and ‘demanding’ to stereotype people with personality disorder diagnoses.” (Stalker et al., 2005)[1]* |
|  |  | *“‘Patients have a hard time trusting a therapist and may only do so after quite some time, which professionals tend to underestimate. These people really need a secure attachment, they fight it for a year, claim help and then reject it again. But if one succeeds in breaking that pattern, one can really mean something.’ On the other hand, a dependency relationship may be perceived as dangerous in community mental health care as many patients become long-term users that lay a large claim on scarce resources, according to the experts.” (Koekkoek et al., 2009)[4]* |
|  |  | *“Clare uses the powerful metaphor of a nursing mother to describe the nature of the relationship. She seems to feel ambivalent about being at the mercy of a new-born baby, whose need to feed on demand cannot be denied. It is as if her capacity to psychologically nourish these clients is being ruthlessly exploited: ‘they latch onto you and it's like suck suck suck suck.’”*  *“…For [clinician], the encounter with the depth of his clients' needs and their sense of emptiness or lack of self ushers in an uncomfortable awareness of his own vulnerability and inner emptiness: ‘What you're met with is a neediness which is bottomless really … and it's almost collateral to the emptiness is the neediness and lack of self … what a lot of borderline patients talk about is being in nothingness, their experiences of nothingness, they have the most acute sense of nothingness that I think you'll ever come across … it's within that that the draining and the exhaustingness of it all, because we all experience emptiness to some degree, but I think these clients … they almost get a heightened sense of all these things … so I almost get a heightened sense of what humanity is and vulnerability.’” (Rizq, 2012)[5]* |
| **Dialectics: Not doing too much or too little** | | |
|  |  | *“Some of the experts are particularly critical of the apparent denial that can be seen to occur in several settings of the long-term nature of the problems of the patient with a severe BPD. That is, a rather naïve and overly “optimistic” attitude characterizes professionals who rapidly discharge such patients. According to the experts, in fact, such optimism is simply “therapeutic nihilism” disguised as optimism… The combination of powerlessness and the blaming of the patient for any lack of progress may result in non-therapeutic behaviors on the part of the professional such as irritation, anger, and even aggression. Less overt but equally destructive is the reduction of the therapeutic encounter to doing as little as possible and simply hoping that a crisis does not arise. Further referral of the patient without substantial justification of the reasons for doing this is another example of really doing nothing. ‘Professionals have many strategies to do completely nothing in therapeutic encounters with the patient.’” (Koekkoek et al., 2009)[4]* |
|  |  | *“One respondent used the term ‘hard to engage services’ to describe what she saw as a model of provision too inflexible to find ways of taking on the often chaotic reality of people’s lives.” (Stalker et al., 2005)[1]* |
|  |  | *“A continuum of severity and complexity was referred to, with acknowledgement that those who were deemed less complex could respond well to routine IAPT treatment but people with what was deemed to be more severe presentations would struggle with routine treatment as they could oscillate from one problem to the next on a weekly basis making adherence to the IAPT model and protocol delivered therapies very challenging to deliver. Participant frustration at the lack of treatment options and the constraints of time-limited therapy was commonly reported.” (Lamph et al., 2019)[6]* |
|  |  | *“In recognition of the heterogeneous needs and capacities of people with PD, most of the pilots set out to provide a range of services. Provision of more than one service or treatment option also enabled most pilots to present a choice to potential service users, a capacity that many believed important in promoting engagement.”*  *“Discharge or disengagement from the service is likely to be difficult and threatening for some service users: it may be viewed as abandonment and may precipitate an increase in behaviour designed to demonstrate need or risk. Some services address this by working toward discharge or self-sufficiency as a specific goal at a specific time from the point of engagement, while others have provisos for re-entry into the service. Some services are developing models for less intensive, ongoing support so that discharge need not be absolute… Some staff of open-ended services felt that there should be a cut-off point, and that allowing ongoing use of a service encourages dependence and reduces motivation and the development of coping strategies for existing clients, while denying others the opportunity of using the service.” (Crawford et al., 2007)[3]* |
|  |  | *“‘Look, although I'm not doing my job properly here — I'm seeing people for longer, I'm, you know, they're dropping in, topping them up every so often when they need it — so on the one hand I see that as a failing in me, but I think it's also a response to the needs of this type of client. So it's not just coincidence, or inexperience in this field, in this type of work with personality disorder.’”*  *“Michael's account exemplifies counsellors' struggle with what appears to be an insoluble paradox — that the establishment of a much-needed therapeutic relationship is precisely what is most likely to evoke yet further trauma for the borderline client when it ends: ‘… they could experience it as a good experience of another person — that not everyone's going to destroy them, if you want, which is their fantasy… But at the same time, you then are faced with the whole thing of ending that. And are you going to do them any good? And are we really just re-traumatising these people again?’”* *(Rizq, 2012)[5]* |
|  | Intervention models | *“I think an ability to manage their emotions better, because they usually come into our service because, on a day to day basis, their emotions are causing them all sorts of difﬁculties in their personal life, with regard to employment, education, leisure activities, and they are perhaps just going from one crisis or problem, to another, and no wonder they are anxious and depressed, which obviously brings them into our world.” (Lamph et al., 2019)[6]* |
|  |  | *“Many relatives have high expectations of the mental health system but are disappointed over time, which also results in a poor relationship between the family of the patient and professionals.” (Koekkoek et al., 2009)[4]* |
|  |  | *“Pilots included several day therapeutic communities: none was residential but they achieved a high degree of consistency through the guidance of shared consultants, staff and service users, and the Association of Therapeutic Communities. A TC is: A safe and secure environment, a place of safety, where people can come and learn how to make relationships… It creates an environment where people engage in normal interactions that trigger behaviours and feelings they have difficulty with: it’s got to be an emotionally safe environment, where they can reflect on and interpret those feelings, so they don’t have adverse consequences.” (Crawford et al. 2007)[3]* |
| **Managing Safety Concerns and Crises: Being Measured and Proactive** | | |
|  |  | *“‘As a CPN if something goes wrong then the buck stops with you and then I think that does not help staff to take positive risks. Staff are very defensive in their practice and very risk adverse and in DBT it is about accepting that this is a risky client group and if we wrap them up in cotton wool all the time that is not treating them and I think it is about having a service that is prepared to take well thought out positive risks and I don’t think we are there yet. Because I think staff are so scared of things going wrong and them getting the blame and being sued it is very hard to allow clients to have some responsibility.’” (Stroud and Parsons, 2013)[7]* |
|  |  | *“There is general recognition that no external agent can stop a person self-harming: responsibility lies with the only person who can change the course of events, the service user themselves. Putting self-harmers into hospital on suicide watch backfires: it takes responsibility away from them. It is better to talk to them about how it comes about and find something to divert them from it.”*  *“Several of the pilot services also have guidelines governing staff–client interaction, such as limits on the amount of time clients can spend in one-to-ones during crises…* *Staff suggest that the act of recording messages has advantages over phone calls because it introduces a slight delay which inhibits impulsiveness and allows a natural pause for consideration. Other services have suggested that e-mail messages to the service have a similar function, even though they will not be read until the next working day…* *Methods for supporting people in crisis developed by pilot services seek to actively involve service users and tend not to provide an instant response. Service providers report that if people have been helped to prepare for crises, a delayed response can help ensure the service user plays an active role in crisis management.” (Crawford et al., 2007)[3]* |
| **Clinician and Wider Service Needs: Whose Needs Are They Anyway?** | | |
|  | Clinician needs | *“Some participants were clear that it was not simply more support that was needed, but a particular type and quality of clinical supervision. Clare was critical of the supervision she was offered in primary care, feeling that it was based on providing expert advice and technical information, rather than examining complex unconscious process issues within the therapeutic relationship. She seems to feel that this is part of a more general tendency where increasingly managed or professionalised forms of practice now take precedence over the emotional aspects of therapeutic work: ‘… it's all about have you filled in the right form, rather than ‘what do you need for your work in terms of emotional support?’”*  *“’I think that people with personality disorder need some kind of secure base if you're going to work with them… I also work in secondary care you see and when you work in secondary care, it's easier to manage people with personality disorder because there's somebody if they do feel suicidal or make a suicide attempt, there's some structure in place. Whereas in primary care you're kind of left on your own with somebody, and you don't have a team to consult, you don't have the support.’” (Rizq, 2012)[5]* |
|  |  | *“‘It actually helps workers to survive in their work, if they have a place to think … One of the main theories about, you know, personality disordered people is that they don’t have the capacity to reflect on themselves and so if [they are involved with] an organization that equally can’t reflect, you’re going to have this sort of mirroring that goes all the way up from the client themselves all the way up through the organization that's trying to help the client.” (Crawford et al., 2010)[8]* |
|  |  | *“Participants noted the flattened hierarchy principle encouraged them to feel that they had a voice in TC and encouraged relational working by bridging the gap between therapists and members: ‘I felt more confident, I felt like I did have a voice in the group.’” (Vyas et al., 2017)[9]* |
|  |  | *“In effect, there are all sorts of expectations on the part of all the players in the system, which may mean that those with the least power are the least likely to have their expectations met.”*  *“Differences in models of understanding might at times be helpful: ‘The opportunity to have a number of disciplines, and talk through a particularly difficult case … that's the strength of the team … it is that exchange of ideas and it does alleviate things an awful lot’… When asked, ‘what helps you keep working with someone when you feel you've tried everything and nothing seems to have helped?’, workers reported that joint working, attention to the needs of workers, including good clinical supervision, trying alternative interventions and working with people who have different models of understanding and alternative perspectives were all potentially helpful… It can also depend on mutual understanding and respect: ‘If you can look at the immediate thing, it might be that you've got different ideas but somewhere above that, the motivation might be common, where the aims overarch.’” (Priest et al., 2011)[10]* |
|  | Interagency working and the wider system | *“The high but inefficient use of the services of several agencies by patients… contributes further to this lack of continuity, the diffused nature of the treatment being offered by professionals, and responsibility for treatment.” (Koekkoek et al., 2009)[4]* |
|  |  | *“‘We get very little feedback from the talk therapies team as to how they felt things went. We obviously get the feedback oh they attended six out of the seven sessions or you know, initially they seem very depressed, but you never get a feedback as to how treatment is going. It’s important I feel because if the service works closely with you it helps you to support the patient better. Added to which many of these patients have other health complaints and better communication can only lead to better treatment.’” (French et al., 2019)[2]* |
|  |  | *“Almost all groups of clinicians reported the need for more training in working with persons who have a personality disorder, particularly for generalist mental health workers and frontline and ancillary staff. Similarly, several groups of clinicians also emphasized the need for a coordinated and cross‐agency approach to training, including staff from other government agencies that have more frequent contact with clients with personality disorders, such as social service organizations. This is in order to encourage an intensive and integrated case management approach (e.g. ‘coordinated whole of team training’, and ‘cross‐agency training within local areas: health, police, community mental health, custodial services, community services’).”*  *“Another theme that emerged was the need for better acknowledgement of the existence of personality disorder as a diagnostic group, and a recognition of the costs and time required to help these clients (e.g. ‘acknowledge the disorder: it exists, is treatable, worthwhile, and economically good to treat’, ‘seeing treatment of personality disorder as core business, alongside mood and psychotic disorders’, and ‘recognition of the enormous cost of the disorder in terms of health service resources, clinician time, [and] administration’).” (Fanaian et al., 2013)[11]* |
|  |  | *“‘Promoting the new service with the right balance of expertise and uncertainty or humility has been challenging. It was difficult to promote the setting up of a specialist service without implying that existing services had somehow failed this client group. It is important to not locate ‘blame’ in either the patient or the worker [who may naturally feel her / himself to be the brunt of criticism]. And it’s a double-edged sword: they want to refer to you, but success is resented: you have to work with that.’” (Crawford et al., 2007)[3]* |
|  | Establishing new services, interventions and skills | *“Clinicians felt their confidence to recall theory and detail post training could fade, as they rolled on with daily case management activity.” (Bosanac et al., 2015)[12]* |
|  |  | *“Several administrators described the importance of ongoing training to accommodate staff turnover, exemplified by the administrator who said ‘There also is no provision for training new people once the training is over. We don't like that [the trainers are] out after that instead of providing training on an ongoing basis.’” (Herschell et al., 2009)[13]* |
|  |  | *“The respondents were asked to provide general feedback on how trainings can address the challenges of implementing DBT mentioned above. Several respondents discussed the difficulties in establishing collaboration between teams at different agencies and viewed this collaboration as key to sustainability of their DBT program, due in part, to the changing of staffing and the loss of many team members due to financial cutbacks.” (Carmel et al., 2014)[14]* |
|  |  | *“Because of the fact the network was initially a pilot there was limited access to funding and resources and their capacity to coordinate care for a large number of clients was restricted. It appeared to the referring bodies that the network was unable to cope with the scale of need.” (Hogard and Ellis, 2010)[15]* |
|  |  | *“Training alone was perceived as insufficient for practice, but a combination of training and hands-on experience was useful to build confidence. One participant stated ‘that could perhaps be a good thing if it was—if everyone saw at least one person through it … they felt comfortable in it, they felt that they could relax … they could actually engage better with the person.’”  (Pigot et al., 2019)[16]* |
|  |  | *“However, there were some clear areas in which the participants were less satisfied, for example, with the viability of post‐training implementation of methods, most notably associated with a perception of an increased time pressure created by some of the practices suggested.” (Thompson et al., 2008)[17]* |

1. Stalker K, Ferguson I, Barclay A. ‘It is a horrible term for someone’: service user and provider perspectives on ‘personality disorder’. *Disability & Society*. 2005;20(4):359-73.

2. French L, Moran P, Wiles N, Kessler D, Turner KM. GPs’ views and experiences of managing patients with personality disorder: a qualitative interview study. *BMJ Open* [Internet]. 2019; 9(2):e026616. DOI: 10.1136/bmjopen-2018-026616

3. Crawford M, Rutter D, Price K, Weaver T, Josson M, Tyrer P, et al. Learning the lessons: a multi-method evaluation of dedicated community-based services for people with personality disorder. *London: National Co-ordinating Centre for NHS Service Delivery & Organisation*. 2007.

4. Koekkoek B, van Meijel B, Schene A, Hutschemaekers G. Clinical Problems in Community Mental Health Care for Patients with Severe Borderline Personality Disorder. *Community Mental Health Journal*. 2009;45(6):508.

5. Rizq R. ‘There's always this sense of failure’: an interpretative phenomenological analysis of primary care counsellors' experiences of working with the borderline client. *Journal of Social Work Practice*. 2012;26(1):31-54.

6. Lamph G, Baker J, Dickinson T, Lovell K. Personality disorder co-morbidity in primary care ‘Improving Access to Psychological Therapy’ services: A qualitative study exploring professionals' perspectives of working with this patient group. *Personality and Mental Health*. 2019;13(3):168-79.

7. Stroud J, Parsons R. Working with borderline personality disorder: A small-scale qualitative investigation into community psychiatric nurses' constructs of borderline personality disorder. *Personality and Mental Health*. 2013;7(3):242-53.

8. Crawford MJ, Adedeji T, Price K, Rutter D. Job Satisfaction and Burnout Among Staff Working in Community-Based Personality Disorder Services. *International Journal of Social Psychiatry*. 2010;56(2):196-206.

9. Vyas A, Spain C, Rawlinson D. Working in a therapeutic community: exploring the impact on staff. *Therapeutic Communities: The International Journal of Therapeutic Communities*. 2017;38(1):32-40.

10. Priest P, Dunn C, Hackett J, Wills K. How can mental health professionals best be supported in working with people who experience significant distress? *Journal of Mental Health* [Internet]. 2011; 20(6):543-54 pp.]. DOI: 10.3109/09638237.2011.577115

11. Fanaian M, Lewis KL, Grenyer BFS. Improving services for people with personality disorders: Views of experienced clinicians. *International Journal of Mental Health Nursing*. 2013;22(5):465-71.

12. Bosanac P, Hamilton B, Beatson J, Trett R, Rao S, Mancuso S, et al. Mentalization-based intervention to recurrent acute presentations and self-harm in a community mental health service setting. *Australasian Psychiatry*. 2015;23(3):277-81.

13. Herschell AD, Kogan JN, Celedonia KL, Gavin JG, Stein BD. Understanding Community Mental Health Administrators' Perspectives on Dialectical Behavior Therapy Implementation. *Psychiatric Services*. 2009;60(7):989-92.

14. Carmel A, Rose ML, Fruzzetti AE. Barriers and solutions to implementing dialectical behavior therapy in a public behavioral health system. *Adm Policy Ment Health*. 2014;41(5):608-14.

15. Hogard E, Ellis R. An evaluation of a managed clinical network for personality disorder: breaking new ground or top dressing? *Journal of Evaluation in Clinical Practice*. 2010;16(6):1147-56.

16. Pigot M, Miller CE, Brockman R, Grenyer BFS. Barriers and facilitators to the implementation of a stepped care intervention for personality disorder in mental health services. *Personality and Mental Health*. 2019;13(4):230-8.

17. Thompson AR, Donnison J, Warnock-Parkes E, Turpin G, Turner J, Kerr IB. Multidisciplinary community mental health team staff's experience of a ‘skills level’ training course in cognitive analytic therapy. *International Journal of Mental Health Nursing*. 2008;17(2):131-7.
