# Supplementary material for: Clinician perspectives on what constitutes good practice in community services for people with complex emotional needs: A qualitative thematic meta-synthesis
Source: PLoS One. 2022 May 5;17(5):e0267787. doi: 10.1371/journal.pone.0267787 (PMC9070883; doi:10.1371/journal.pone.0267787)
Supplement: S2 Appendix — (DOCX) [file pone.0267787.s005.docx]

**S2 Appendix – Detailed Eligibility Criteria**

- *Population (diagnosis)*: Clinicians should discuss their perspectives of services for people with a diagnosis of ‘personality disorder’ or related symptoms/diagnoses. Related symptoms include repeated self-harm or suicide attempts, complex trauma or complex PTSD, and emotional dysregulation or instability (We acknowledge this list is not comprehensive). The primary diagnosis or focus of treatment of the sample should be personality disorder or similar. Treatments that are primarily for other conditions are not included. This excludes qualitative studies of specialist treatment services for other diagnoses including substance misuse conditions.
- *Population (clinicians)*: Relevant stakeholder groups include psychiatrists, psychiatric nurses, psychologists, general practitioners, peer support workers, service managers, commissioners and other professionals who work with people who have received a diagnosis of “personality disorder” or have related symptoms or needs.
- *Settings*: Community services providing mental health support for people who have been diagnosed with a “personality disorder” or experience symptoms or needs which have been associated with “personality disorder”.

Services can be a specialist ‘personality disorder’ service or a generic mental health/primary care/community service, as long as there is some reference to care for people with a diagnosis of personality disorder or related needs. For example, an eligible paper could describe initiatives within generic mental health/primary care service which focus on better meeting the needs of this group of people or describe the experiences of this group of people receiving generic services.

Services for young people will be excluded unless they are transitioning to adult services. For example, qualitative data of people’s experiences of CAMHS treatment per se will be excluded. But data from people’s experiences of moving from CAMHS to adult services will be included.

Hospital services, such as day hospital treatments, are included provided participants still reside in the community through the course of treatment. Participants recruited from inpatient settings may be included provided the treatment being evaluated is a community/outpatient treatment to which they have transitioned. Otherwise, inpatient and crisis services are excluded, as are forensic services and services specifically for offenders.

- *Limits:* Studies published since 2003 will be eligible. 2003 marked a change in UK policy for “personality disorder” services with the publication of implementation guidance for the development of services 'Personality Disorder: No longer a diagnosis of exclusion'. (NIMHE, 2003)
- *Design*: Data should be qualitative and analysed using a recognised qualitative method (such as thematic analysis). Written data from questionnaires can be included if the data are analysed using a recognised qualitative method.

Data from trials and other quantitative studies will be included if the study is naturalistic (i.e. offers a similar delivery/support to a community mental health team or specialist service, e.g. a good quality DBT treatment delivered by mental health practitioners embedded in a mental health service).

- A pragmatic decision was made to exclude papers that had not been peer-reviewed, were not in English, and dissertations or theses.

**References**

National Institute for Mental Health for England. *Personality Disorder: No Longer a Diagnosis of Exclusion. Policy Implementation Guidance for the Development of Services for People with Personality Disorder*. London: NIMH(E); 2003.
