# Supplementary material for: Clinician perspectives on what constitutes good practice in community services for people with complex emotional needs: A qualitative thematic meta-synthesis
Source: PLoS One. 2022 May 5;17(5):e0267787. doi: 10.1371/journal.pone.0267787 (PMC9070883; doi:10.1371/journal.pone.0267787)
Supplement: Attachment — Submitted filename: Response to Reviewers v2.docx [file pone.0267787.s006.docx]

Dear PLOSONE

Re: [PONE-D-20-39422R1]

Thank you for the invitation to revise and resubmit our manuscript **“**Clinician perspectives on what constitutes good practice in community services for people with Complex Emotional Needs: A qualitative thematic meta-synthesis”, in response to the valuable editorial and reviewer comments. We have detailed our responses to each point, and the changes made to the manuscript, below.

We hope that the manuscript is now suitable for publication in PLOS ONE and look forward to hearing from you further.

Yours sincerely,

Luke Sheridan Rains, Sonia Johnson, Jordan Troup, Billie Lever Taylor, Eva Broeckelmann, Jessica Russell, Tamar Jeynes, Chris Cooper, Thomas Steare, Zainab Dedat, Shirley McNicholas, Sian Oram, and Oliver Dale.

***Editorial comments from 19/11/2021***

*1. We note that several of your files are duplicated on your submission. Please remove any unnecessary or old files from your revision, and make sure that only those relevant to the current version of the manuscript are included.*

Response – we have now removed the duplicate files

*2. Thank you for stating "Funding" section of your manuscript:*

*"This paper presents independent research commissioned and funded by the National Institute for Health Research (NIHR) Policy Research Programme, conducted by the NIHR Policy Research Unit (PRU) in Mental Health (grant no. PR-PRU-0916-22003). The views expressed are those of the authors and not necessarily those of the NIHR, the Department of Health and Social Care or its arm’s 591 length bodies, or other government departments. The funders had no role in study design, data collection and analysis, decision to publish, or preparation of the manuscript."*

*We note that you have provided funding information that is not currently declared in your Funding Statement. However, funding information should not appear in the Acknowledgments section or other areas of your manuscript. We will only publish funding information present in the Funding Statement section of the online submission form.*

*Please remove any funding-related text from the manuscript and let us know how you would like to update your Funding Statement. Currently, your Funding Statement reads as follows:*

*"This paper presents independent research commissioned and funded by the National Institute for Health Research (NIHR) Policy Research Programme, conducted by the NIHR Policy Research Unit (PRU) in Mental Health (grant no. PR-PRU-0916-22003). The views expressed are those of the authors and not necessarily those of the NIHR, the Department of Health and Social Care or its arm’s 591 length bodies, or other government departments. The funders had no role in study design, data collection and analysis, decision to publish, or preparation of the manuscript."*

*Please include your amended statements within your cover letter; we will change the online submission form on your behalf.*

Response - We have now removed the funding information from the manuscript and included it in our cover letter as requested. This statement is tracked in the cover letter to make it clear how the letter has changed since our original submission. The funding statement that you provided is correct and to eliminate ambiguity is repeated below:

"This paper presents independent research commissioned and funded by the National Institute for Health Research (NIHR) Policy Research Programme, conducted by the NIHR Policy Research Unit (PRU) in Mental Health (grant no. PR-PRU-0916-22003). The views expressed are those of the authors and not necessarily those of the NIHR, the Department of Health and Social Care or its arm’s 591 length bodies, or other government departments. The funders had no role in study design, data collection and analysis, decision to publish, or preparation of the manuscript."
